# A colonic mass revealing a disseminated crystal storing histiocytosis secondary to indolent multiple myeloma: a case report with literature review

**DOI:** 10.1186/s12876-020-01364-2

**Published:** 2020-07-31

**Authors:** Adrien Contejean, Frédérique Larousserie, Didier Bouscary, Anthony Dohan, Bénédicte Deau-Fischer, Tali-Anne Szwebel, Marion Dhooge, Benoit Terris, Marguerite Vignon

**Affiliations:** 1 Haematology Department, AP-HP, Cochin University Hospital, 27 rue du Faubourg Saint Jacques, Paris, France; 2 Paris University, Paris, France; 3 Pathology Department, AP-HP, Cochin University Hospital, Paris, France; 4 Radiology A Department, AP-HP, Cochin University Hospital, Paris, France; 5 Internal medicine Department, AP-HP, Cochin University Hospital, Paris, France; 6 Gastroenterology Department, AP-HP, Cochin University Hospital, Paris, France

**Keywords:** Cristal storing histiocytosis, Multiple myeloma, Daratumumab, Case report

## Abstract

**Background:**

Crystal storing histiocytosis is a rare disorder associated with monoclonal gammopathy. In this disease, monoclonal heavy and light chains accumulate in the lysosome of macrophages, leading to histiocytic reaction in different organs. It is secondary to the presence of a small B-cell clone, responsible for monoclonal immunoglobulin production. Histological diagnosis is a challenge and differential diagnoses include fibroblastic and histiocytic neoplasm. Clinical manifestations depend on the involved organs, rarely including peritoneum or digestive tract.

**Case presentation:**

We present a case of a 75-year-old with a medical history of colonic carcinoma. She presented with abdominal pain and inflammatory syndrome revealing a colonic mass. Hemicolectomy was performed. Initial diagnosis was fibroblastic tumour. The patient worsened, and diagnosis of a diffuse crystal storing histiocytosis was finally done. Haematological exploration found an indolent IgG-kappa multiple myeloma. The initial treatment with conventional chemotherapy did not permit an improvement of the patient condition. Immunotherapy with anti-CD38 monoclonal antibody (daratumumab) was proposed with a clinical and biological response.

**Conclusion:**

This case report emphasizes the histopathological challenge of histiocytic tumours which may involve digestive track. It focuses on the concept of monoclonal gammopathy of clinical significance, which can have a large spectrum of manifestations.

## Background

Crystal storing histiocytosis (CSH) is a rare disorder associated with monoclonal gammopathy. It is defined by accumulation of monoclonal heavy and light chains in the lysosome of macrophages. CSH may result in a variety of clinical manifestations depending on the involved organs, but digestive tract involvement is rare.

## Case presentation

**Fig. 1:**
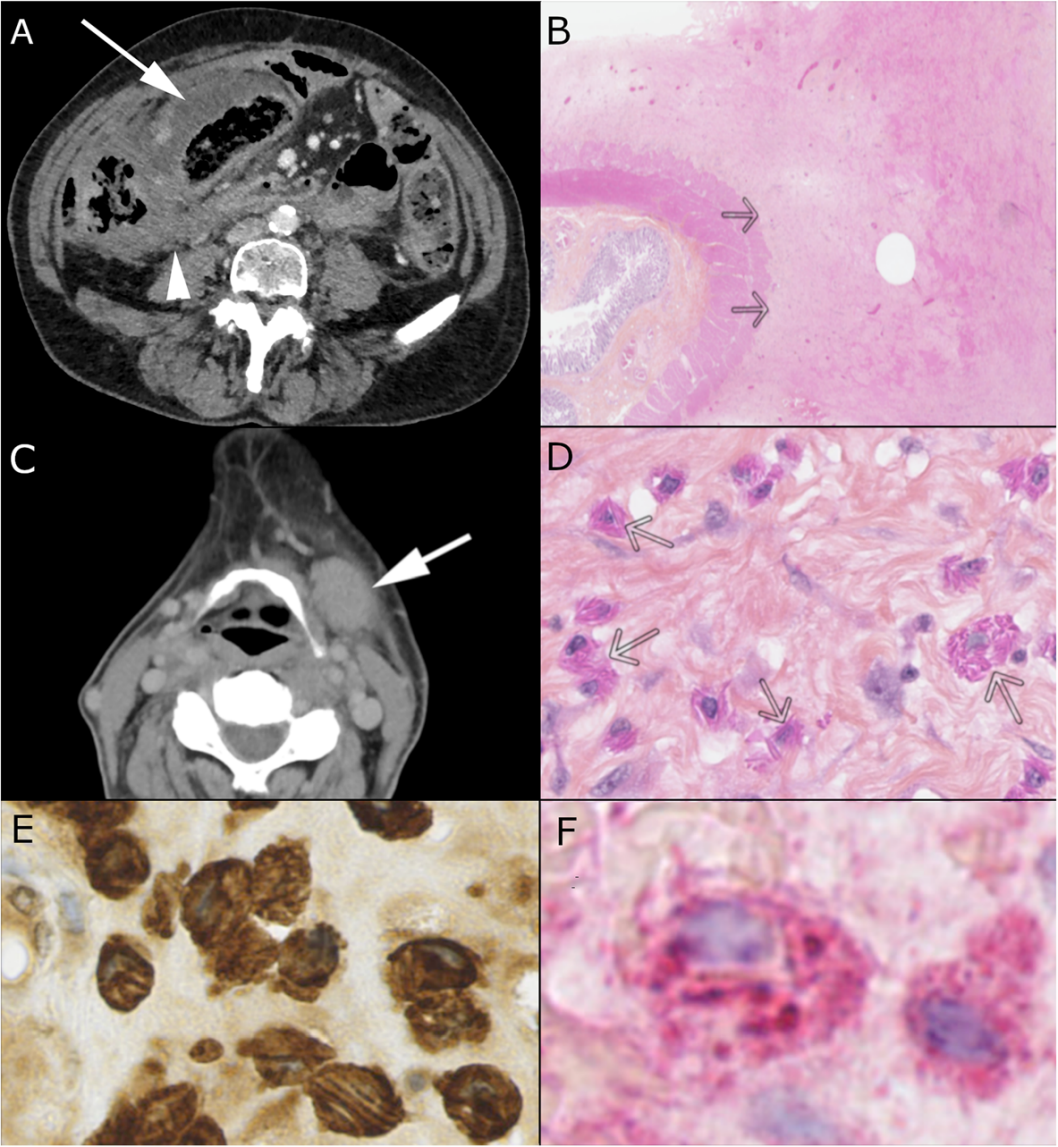
CT-scan and histological analyses. **a**: Axial section of abdominal CT-scan showing a pseudotumoral thickening of the right colonic wall (arrow) with diffuse peritoneal effusion (arrowhead). **b**: Right colectomy showing a tumoral proliferation developed from subserosal part of the bowel without infiltration of muscularis. **c**: Axial section of a cervical CT-scan showing a left sub-mandibular lymphadenopathy of 30 mm in diameter (arrow). **d**: The tumour comprised numerous histiocytes with crystals in eosinophilic cytoplasm (arrows). **e**: Histiocytes are positive for CD163. **f**: Crystals are positive for the kappa light chain

A 75-year-old woman presented with abdominal pain and altered general condition. Her medical history was left colectomy for localised colonic adenocarcinoma 12 years ago, radiotherapy for anal epidermoid carcinoma diagnosed on a histologic piece of hemorrhoidectomy 3 years ago, type II diabetes, and IgG-kappa monoclonal gammopathy. She reported an abdominal discomfort for more than 1 year then weight loss in the past 6 months. These symptoms were explored a year ago with CT-scan and colonoscopy that were normal. At admission, CT-scan showed a pseudotumoural thickening of the right colonic wall with diffuse peritoneal effusion (Fig. 1A) but the colonoscopy without biopsies revealed no mucosal abnormalities. A surgical exploration was decided: per-operative examination revealed a mass infiltrating the right colon, the caecum, and the peritoneum leading the surgeon to perform a right hemicolectomy. First histological conclusion (Fig. 1B) was desmoid-type abdominal fibromatosis and after review fibroblastic/myofibroblastic tumour of intermediate malignancy. No treatment was proposed, and the patient was addressed to our institution.

The patient’s condition worsened within 6 months with a loss of 12 kg, diarrhoea, abdominal pain a slowing down of her intestinal tract and dysphagia, without vomiting. Clinical examination showed a sub-mandibular tumefaction which was confirmed on a CT-scan (Fig. 1C). Abdominal imaging showed peritoneal effusion and multiples nodules suggesting carcinosis. Biopsy of the sub-mandibular mass showed numerous histiocytes with intracytoplasmic eosinophilic crystals, engulfed within a dense reactional fibroblastic population (Fig. 1D). Those findings allowed a diagnosis of CSH. Immunohistochemistry confirmed macrophagic nature of the cell infiltrate, positive for CD163 (Fig. 1E). Crystals were stained with an anti-kappa antibody (Fig. 1F) and not with an anti-lambda antibody, consistent with a monoclonal light chain. The review of the colonic mass histology confirmed the diagnosis and allowed us to classify it as a generalized CSH [1].

Haematological explorations detected a monoclonal IgG-kappa at 26 g/l and imbalanced kappa/lambda free light chains (Tables 1). Serum creatinine and calcium were normal. Haemoglobin level was 10.8 g/dL. A bone marrow aspiration found 16% of dysmorphic plasma cells. Cytogenetics identified a t(11;14) translocation. PET-scan was normal. A diagnosis of indolent IgG-kappa multiple myeloma (MM) complicated with diffuse CSH was made. A first line treatment based on proteasome inhibitor was proposed with bortezomib (1.3 mg/m2 D1;D4;D8;D11), cyclophosphamide (350 mg/m2 D1;D8;D15) and dexamethasone (40 mg D1-D4). After three cycles, the general status of the patient worsened with persistent abdominal pain and a marked inflammatory syndrome. Introduction of continuous corticosteroid therapy with prednisone at 0.8 mg/kg/d improved the abdominal pain.

**Table 1:** Biological results at baseline and under treatment

	Diagnosis	After C3 Bor-Cyc-Dex	After C3 Dara-Len-Dex	After C6 Dara-Len-Dex
Monoclonal spike (g/L)	26	23	5	3.3
Free light chains kappa/lambda (mg/L)	54/7	107/3	29/1	27.1/1.4
Hemoglobin (g/dL)	10.8	10.3	12	11.8
Serum calcium (mmol/L)	2.3	2.03	2.21	2.33
Serum creatinine (μmol/L)	73	75	88	85
Albumin (g/L)	27	26	30	40
CRP (mmol/L)	85	106	2	1

*Bor-Cyc-Dex* Bortezomib Cyclophosphamide Dexamethasone; *CRP C-Reactive Protein*; *Dara-Len-Dex* Daratumumab Lenalidomide Dexamethasone

A second-line regimen based on immunodulatory agents and monoclonal antibody was done, with daratumumab (1400 mg), lenalidomide (25 mg D1-D21) and dexamethasone (40 mg/week). Partial response (PR), defined by reduction of 50% of gammopathy level, was obtained (Table 1) with significant improvement of the patient’s condition. Although abdominal CT-scan showed persistence of peritoneal nodules, we noticed a disappearance of peritoneal effusion. After 6 months of treatment, immunochemical PR persisted and albumin normalized (Table 1). Medullar biopsy was normal. Unfortunately, 2 months later a mechanical occlusion of the intestine with perforation occurred. The evolution was rapidly fatal with multiple organ failure syndrome and death of the patient despite intensive care and surgical management.

## Discussion and conclusions

Monoclonal gammopathies always result from B-cell clones and can be related to MM or lympho-plasmocytic lymphoma. Sometimes the B-clone is quiescent, but organ damage can occur due to the toxicity of the monoclonal immunoglobulin itself, or by other mechanisms. Thus the concept of monoclonal gammopathy with clinical significance (MGCS) was introduced [2]. Most MGCS-associated lesions are caused by the deposition of entire or parts of the monoclonal immunoglobulins. Crystalline deposits are present in three distinct entities: acquired Fanconi syndrome, crystalline keratopathy and CSH. We must make a distinction between localized CSH, involving one organ system, often in the head and neck region (35%) and diffuse CSH, involving two or more distant organ sites [1]: bone marrow (97%), liver (47%), spleen (44%) and lymph nodes (44%) which are the most frequent. Digestive tract involvement is rare. Inflammatory syndrome may occur during generalized CSH.

In CSH, light chain is almost always kappa, suggesting that occurrence of CSH is mainly linked to structural characteristics of the monoclonal immunoglobulin. Plasma cells produce a structurally aberrant immunoglobulin which aggregates in crystals accumulated in the lysosome of macrophages because of proteolysis resistance [3]. The mechanism that promotes crystallization of protein and that affects intra-lysosomal degradation remains unclear.

The diagnosis of CSH represents a clinical and histopathological challenge, especially in peritoneal and digestive tract involvement where peritoneal carcinosis may be wrongly suggested. Characterization of histiocytes with abundant crystalline inclusions is the main feature of CSH [4]. Cytologically benign histiocytes contain eosinophilic crystals that distend their cytoplasm. Immunohistochemistry demonstrates intra-cytoplasmic inclusions made of monotypic light and/or heavy chains of immunoglobulins. There are numerous differential diagnoses of histiocytic reaction. In our case a diagnosis of fibroblastic tumour was initially done.

In a review, 23 cases of generalized CSH among a total of 131 CSH cases were identified [5]. Their prognosis is worse because of organ impairment. As in other MGCS, treatments recommendation is to target the underlying malignancy to stop the production of the monoclonal immunoglobulin [6]. However, despite haematological response, the clearing of histiocytic lesions is inconsistent. Between 2000 and 2019, six detailed cases of generalized CSH treated in the era of novel agents have been published (Table 2). CSH was diffuse and involved kidney (*n* = 2), bone marrow and spleen (*n* = 2), mesenteric panniculitis (*n* = 1), and lung (*n* = 1). Three patients had a MM [2, 3, 13] two a lymphoplasmacytic lymphoma [5, 6] and one monoclonal gammopathy [7]. Light chain was always kappa (IgG [3], IgA [1], IgM [1] or LC only [1]). Bortezomib base therapy was used in five of them and rituximab in one case. Four patients obtained a very good partial response and two patients obtained a PR. In 3/6 cases haematological response did not stop organ impairment and patients evolved to end stage renal failure (*n* = 2) or pancytopenia (*n* = 1). In one case patient general status improved but mesenteric lymph nodes were still present. Two cases had a complete organ response.

**Table 2:** Summary of published case reports of generalized CSH treated in the era of novel agents

Reference	date of publication	Age	Sex	Underlying disease	Organ involvment	Evolution before diagnosis	Ig	Treatment	Haematological response	Organ response	Evolution
Boudhabhay [7]	2018	60	M	multiple myeloma	cornea, kidney	2 years	IgGkappa	bortezomib-lenalidomide-dexamethasone	PR	NO	End stage renal failure
Wu [8]	2017	48	M	multiple myeloma	kidney	Unknown	IgGkappa	bortezomib-cyclophosphamide-dexamethasone	VGPR	NO	End stage renal failure
Aline-Fardin[9]	2015	69	M	monoclonal gammopathy	mesenteric panniculitis, lympho node, kidney	6 months	LC kappa	bortezomib-DXM	VGPR	YES	Persistent remission (12 months)
Baird [10]	2015	53	F	lymphoplasmocytic lymphoma	lung, mediastinal lymph node, bone marrow	18 months	IgM lambda	bortezomib-cyclophosphamide-dexamethasone-rituximab	VGPR	YES	Remission
Hu [11]	2012	48	F	multiple myeloma	spleen, bone marrow	2 years	IgGkappa	bortezomib-thalidomide-dexamethasone-rituximab	VGPR	YES	Remission
Robak [12]	2002	54	M	lymphoplasmocytic lymphoma	spleen, bone marrow	1 year	IgAkappa	ritixmab	PR	NO	Pancytopenia

*F* Female; *M* Male; *PR* Partial response; *VGPR* Very good partial response

Here**,** we describe the first case of a patient with CSH treated with daratumumab-based therapy. Daratumumab is a novel targeted anti-CD38 monoclonal antibody that is being increasingly used in the treatment of MM. In a relapse setting, the association of daratumumab with lenalidomide and dexamethasone permits an overall response rate of 92% in patients with MM [13]. In the context of AL amyloidosis, daratumumab can be used in frail patients with promising results [14]. Thus, immunotherapy in the management of MGCS seems to have an increasing role by improving the control of toxic immunoglobulin production. A better molecular understanding of disease may help to define the optimal treatment.

## Data Availability

Not applicable.

## Abbreviations

CSH: Crystal Storing Histiocytosis
MGCS: Monoclonal Gammopathy with Clinical Significance
MM: Multiple Myeloma
PR: Partial Response
